# Increased prevalence of deep vein thrombosis and mortality in patients with Covid-19 at a referral center in Brazil

**DOI:** 10.1177/02683555211041931

**Published:** 2021-09-08

**Authors:** Jose Maria Pereira de Godoy, Gleison Juliano da Silva Russeff, Carolina Hungaro Cunha, Debora Yuri Sato, Desirée Franccini Del Frari Silva, Henrique Jose Pereira de Godoy, Mariana Orate Menezes da Silva, Henrique Amorim, Marina Morães Lopes Soares, Maria de Fatima Guerreiro Godoy

**Affiliations:** 1 Cardiology and Cardiovascular Surgery in Medicine School of Sao Jose do Rio Preto-FAMERP and CNPq (National Council for Research and Development), Brazil; 2 Vascular Surgery of Service Ecography in Hospital de Base-Medicine School of Sao Jose do Rio Preto-FAMERP-Brazil; 3 Department General Surgery of the Medicine School in São José do Rio Preto-FAMERP-Brazil and Member Research Group in the Clínica Godoy, Sao Jose do Rio Preto, Brazil; 4 Vascular Surgery Service in Medicine School in Sao Jose do Rio Preto-FAMERP-Brazil; 5 Stricto Sensu in Medicine School in São José do Rio Preto-FAMERP; Clínica Godoy, Sao Jose do Rio Preto, Brazil

**Keywords:** Prevalence, deep vein, thrombosis, mortality, Covid-19

## Abstract

**Background:**

Among the multiple complex pathophysiological mechanisms underlying Covid-19 pneumonia, immunothrombosis has been shown to play a key role.

**Objective:**

The aim of the present study was to assess the monthly prevalence of deep venous thrombosis in a university hospital that admitted 5159 patients with Covid-19 in the medical ward and intensive care unit (ICU) and investigate whether there has been an increase in the prevalence of deep vein thrombosis and dead recently.

**Method:**

A clinical trial was conducted evaluating 5159 patients admitted to the university hospital, Hospital de Base in São Jose do Rio Preto-Brazil, with a positive test for Covid-19, the prevalence of monthly deep venous thrombosis and the increase in thrombotic and events and mortality in March 2020 to April 2021 compared to the previous January and February with March–April of 2021. The evaluated by Fisher's exact test.

**Results:**

The prevalence of deep vein thrombosis varied between the months of 0.26% to 7%, with an average of 2.5%. The months of March and April 2021 had a significant increase in venous thrombosis and mortality in relation to the months of January and February 2021.

**Conclusion:**

The prevalence of deep venous thrombosis was variable during the months evaluated, since the beginning of Covid-19, but there was a significant increase in these last two months.

## Introduction

Among the multiple complex pathophysiological mechanisms underlying Covid-19 pneumonia, immunothrombosis has been shown to play a key role. One of the most dangerous consequences of prothrombotic imbalance is the increased incidence of micro- and macro-thrombotic phenomena, especially deep vein thrombosis (DVT) and pulmonary embolism (PE).^1^

Platelets and the coagulation cascade are effectively targeted by antithrombotic approaches, which present an inherent risk of bleeding. In addition, antithrombotics cannot completely prevent thrombotic events, implying a therapeutic gap due to a third mechanism not yet adequately addressed, namely, inflammation.^2^

The results of the last decade have revealed a sophisticated connection between innate immunity, platelet activation and coagulation, called immunothrombosis, where inflammation and thrombosis are strongly connected processes that contribute to contain the spread of the pathogen in an effector defense mechanism of the host called immunothrombosis.^3^

The study evaluated 87 patients admitted to the ward or ICU with duplex, detected a DVT in 4 (4.5%) patients (3 femoral, 1 popliteal), of which 1 there was no prevalence of prophylaxis with low molecular weight heparin (MRPA). 21 pulmonary angiographies were performed by CT, being positive for PE in 5 cases (23.8%); only 2 patients suffered DVT.^4^ Study identifies that an incidence of DVT of the proximal lower limb in a patient with Covid-19 was 3%.^5^ Systematic screening for DVT in patients admitted to the ICU was not associated with a higher diagnosis of VTE or a reduced diagnosis of EP.^6^ The aim of the present study was to assess the monthly prevalence of deep venous thrombosis in a university hospital that admitted 5159 patients with Covid-19 in the medical wart and intensive care unit (ICU) and investigate whether there has been an increase in the prevalence of deep vein thrombosis and dead recently.

## Material and method

### Casuistic and location

All patients with Covid-19 positive admitted to university hospital- Hospital de Base in São Jose do Rio Preto-Brazil, were evaluated from March 2020 to April 2021.

### Design

A clinical trial was conducted evaluating all patients admitted to the Hospital de Base in São Jose do Rio Preto-SP-Brazil, with a positive test for Covid-19, the prevalence of monthly deep venous thrombosis and the increase in thrombotic and events and mortality in these two months compared to the previous two months, assessed by Fisher's exact test.

### Inclusion criteria

Patients with positive tests and who were indicated to receive oxygen therapy, severe pulmonary symptoms, and associated infections or other diseases that contributed to the worsening of the clinical condition.

### Exclusion criteria

All patients tested positive for Covid-19 and who had mild signs and symptoms and did not require oxygen therapy and other associated hospital measures.

### Ethical consideration

The study was approved by the Research Ethics Committee Medicine School of São Jose do Rio Preto-Brazil#4.720.521.

### Statistical consideration

Descriptive statistician of the data and Fisher's exact test were performed, considering an alpha error of 5%.

### Development

All patients admitted to the medical ward and intensive care unit (ICU) of the Hospital de Base-São Jose do Rio Preto-Brazil were evaluated from March 2020 to April 2021 with suspicion of Covid-19 and evaluated for their positivity by the RT-PCR test. These data were entered into Excel tables and analyzed using the Static direct 3 statistical program. Positive patients were admitted according to severity in the medical ward and intensive care unit (ICU). All patients received routine prophylactic anticoagulation and in this study the monthly, average prevalence and whether there was a period of higher prevalence of deep venous thrombosis and mortality were analyzed.

### Results

**Figure 1.**
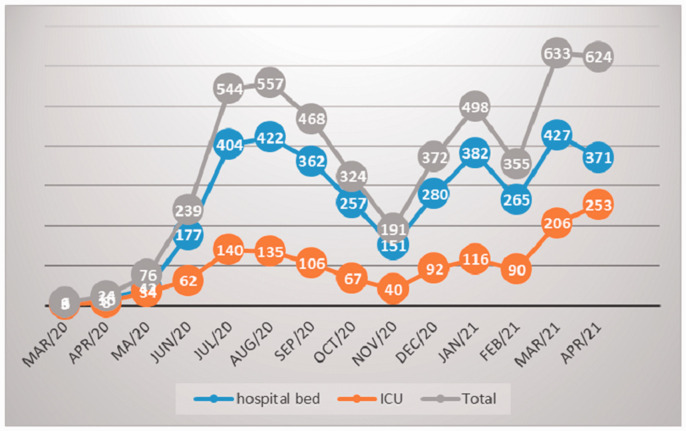
Evaluation of the total number of patients admitted to the ward and ICU with Covid-19.

Had 8108 patients with suspected Covid-19 were hospitalized, 6277 in the medical ward and 1831 in the intensive care unit at the Hospital de Base in São Jose do Rio Preto, March 2020 to April 2021, Table 1. Of these, 5159 patients with Covid-19 were confirmed, 3706 in the medical ward and 1453 in the intensive care unit, as shown in Table 2 and Figure 1.

**Table 1.** Total patients with suspected Covid-19 (regardless of results).

Month/year	Medical ward	Intensive care unit	Total
March/20	50	16	66
April/20	110	28	138
May/20	177	55	232
June/20	386	103	489
July/20	638	185	823
August/20	650	158	808
September/20	611	142	753
October/20	578	113	691
November/20	366	68	434
December/20	471	123	594
January/21	571	137	708
February/21	439	116	555
March/21	526	214	740
April/21	503	269	772
May/21	201	104	305
Total	6277	1831	8108

**Table 2.** Total interned (confirmed COVID^a^).

Month/year	Medical ward	Intensive care unit	Total
March/20	3	3	6
April/20	16	8	24
May/20	42	34	76
June/20	177	62	239
July/20	404	140	544
August/20	422	135	557
September/20	362	106	468
October/20	257	67	324
November/20	151	40	191
December/20	280	92	372
January/21	382	116	498
February/21	265	90	355
March/21	427	206	633
April/21	371	253	624
May/21	147	101	248
Total	3706	1453	5159

**Figure 2.**
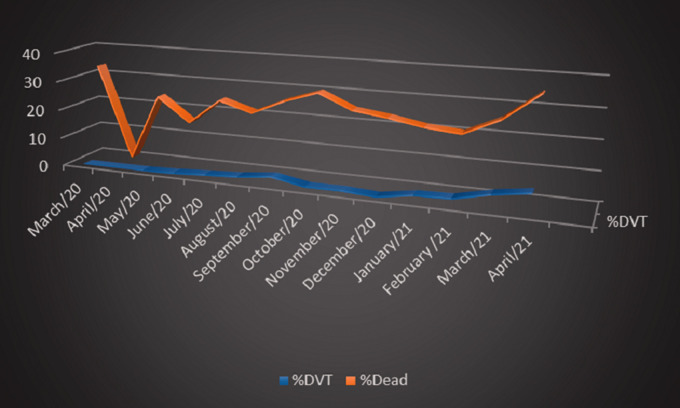
Variations in monthly prevalence’s of deep vein thrombosis and mortality.

The prevalence of deep vein thrombosis varied between the months of 0.26% to 7%, with an average of 2.5% in this period, as shown in Table 3. The months of March and April 2021 had a significant increase in venous thrombosis and mortality in relation to the months of January and February 2021, Fisher's exact test p-value = 0.0001. Figure 1 illustrate the variations in the number of patients total and hospitalized in the medical ward and ICU and Figure 2 shows the variations in monthly prevalence’s of deep vein thrombosis and mortality

**Table 3.** Number of patients with Covid-19 admitted to the infirmary and ICU of Hospital de Base-Brazil, number of positive DVT patients per month and the prevalence of this occurrence, deaths and monthly percentage.

Month/year	Medical ward	Intensive care unit	Total	DVT/Covid/+	%	Dead	%
March/20	3	3	6	–	–	2	33,33
April/20	16	8	24	–	–	12	0,5
May/20	42	34	76	–	–	18	23,68
June/20	177	62	239	1	0,41	37	15,4
July/20	404	140	544	6	1,1	132	24,2
August/20	422	135	557	10	1,7	116	20,8
September/20	362	106	468	14	2,99	121	25,8
October/20	257	67	324	3	0,92	96	29,6
November/20	151	40	191	2	1,0	47	24,6
December/20	280	92	372	1	0,26	86	23,11
January/21	382	116	498	11	2,2	105	21,0
February /21	265	90	355	9	2,5	71	20,0
March/21	427	206	633	33	5,2	162	25,5
April/21	371	253	624	44	7,0	217	34,7
Total	3706	1453	5159	134	2,5	1212	23,49

### Discussion

The present study confirms a hypothesis that the involvement with venous thrombotic and mortality conditions increased in the months of March and April in relation to the previous months. The Hospital de Base is a teaching hospital that offered 117 to 197 ICU beds in that period and 280 beds in the ward for Covid-19 patients. The average prevalence of DVT was around 2.5%, but there is a significant variation of 0.26 to 7% in this period. In the first three months of Covid-19 in March, April and May, there was no diagnosis of deep vein thrombosis in these patients. December was one of the months with the lowest number of patients diagnosed with DVT, however a logical explanation was not detected.

The significant increase in incidence has caught our attention in recent months, a fact that led to this study. Another aspect that alerted us was the characteristics of the thrombosis sites, where unusual occurrences such as bilateral soleus veins, a higher incidence of thrombotic event bilaterally. A more detailed investigation of these thrombotic events is being carried out.

Several factors should be considered regarding the actual prevalence of DVT in patients with Covid-19 and analyzing patients in the ward and ICU separately. However, difficulties in diagnosis make this prevalence underdiagnosed. Negative factor in patients in the ICU is the fact that they are bedridden and the presence of edema is not always observed. Standing patients in the infirmary provide the appearance of edema.

The D-dimer test is an important tool that allows you to alert the doctor about its occurrence. Thus, a constant screening is necessary, but the most prevalent thrombotic site is the lungs. The prophylaxis of the major cause of mortality in Covid 19, which is pulmonary thrombosis, is done using antiplatelet and anticoagulant drugs, but immunothrombosis is a recent concept associated with thrombosis and inflammatory processes.

In the prophylaxis of immunothrombosis it does not always have the same results in relation to conventional thrombosis. We had late failures of post-Covid-19 anticoagulation and that is one of the challenges we are facing. The anticoagulant associated with low-dose aspirin is an option that we use to prevent abortion in patients with antiphospholipid antibody syndrome, in these cases improving abortion prophylaxis.^7,8^ Therefore, further research should be carried out to evaluate the best prophylactic option for these patients. At the moment, the association of low-dose aspirin with prophylactic anticoagulation is the most suggested, however the risks and benefits must be evaluated.

## Conclusion

The prevalence of deep venous thrombosis was variable during the months evaluated, since the beginning of Covid-19, but there was a significant increase in these last two months.
